# Syngas Production in a 1.5 kW_th_ Biomass Chemical Looping Gasification Unit Using Fe and Mn Ores as the Oxygen Carrier

**DOI:** 10.1021/acs.energyfuels.1c01878

**Published:** 2021-08-17

**Authors:** Oscar Condori, Luis Francisco de Diego, Francisco Garcia-Labiano, María Teresa Izquierdo, Alberto Abad, Juan Adánez

**Affiliations:** Department of Energy and Environment, Instituto de Carboquímica (ICB)−Consejo Superior de Investigaciones Científicas (CSIC). Miguel Luesma Castán 4, 50018 Zaragoza, Spain

## Abstract

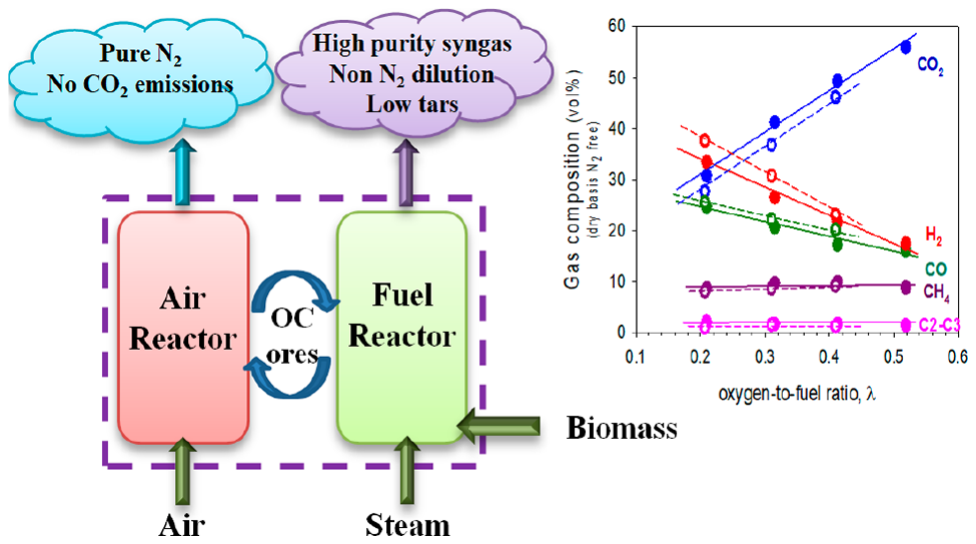

Biomass chemical looping gasification (BCLG) uses lattice oxygen from an oxygen carrier instead of gaseous oxygen for high-quality syngas production without CO_2_ emissions. In this work, the effect of the main operating variables, such as oxygen/biomass ratio (λ), gasification temperature, and steam/biomass ratio (S/B), was investigated using two low-cost materials: a Fe ore and a Mn ore. Oxygen fed to the air reactor for oxidation was used as an effective method for controlling the amount of lattice oxygen used for syngas production. The main variable that affected the process performance and the syngas quality was λ, while the fuel reactor temperature and the S/B ratio had a minor effect. Small performance differences found between the ores can be attributed to different degrees of CH_4_ and light hydrocarbons reforming in the process. The CO_2_ content in the syngas was high (40 −43%) under autothermal conditions because the gasification reactions required the heat to be generated by combustion. CH_4_ contents of around 10% were found in syngas, coming from the unburned or unreformed volatiles. Syngas yields around 0.60 Nm^3^/kg of dry biomass were found for both ores. Additionally, high biomass conversions (*X*_b_ > 94%) and carbon conversion efficiencies (η_cc_ > 95%) were obtained in all cases, showing the capability of the process of avoiding CO_2_ emissions to the atmosphere. No agglomeration was found in the bed during the BCLG process, although attrition rates were high, leading to lifetimes of 160 and 300 h for the manganese and iron ores, respectively. Migration of Fe or Mn to the external part of the particle, generating a metal concentrated shell, was observed. Its detachment was responsible for the decrease in the oxygen transport capacity (*R*_OC_) of the material with the operating time and the reduced lifetime. The results obtained here allowed the iron ore to be considered as an oxygen carrier suitable for the BCLG process.

## 1 Introduction

The current situation on climate change and global warming, associated with excessive energy consumption by humans, has promoted scientific efforts in the investigation of processes that can replace the use of fossil fuels with renewable energy sources, reducing emissions of greenhouse gases (GHGs).^1^ Additionally, one of the main objectives implemented by the Paris Agreement on climate change is to produce biofuels from renewable sources to replace fossil fuels and mitigate GHG emissions.

In this sense, gasification processes have been widely studied at different scales recently, with the main objective of converting fuels (coal, biomass, heavy condensable hydrocarbons, etc.) into synthesis gas for the production of energy, biofuels (via Fischer–Tropsch synthesis), or chemicals.^2,3^

Biomass is an abundant renewable energy source on Earth that removes CO_2_ from the atmosphere during its growth through photosynthesis. If CO_2_ formed during combustion is captured and then stored [bioenergy with carbon capture storage (BECCS) technologies], it is possible to achieve net-negative CO_2_ emissions into the atmosphere.^4,5^

Biomass gasification is a thermochemical conversion process, which converts biomass into high-energy fuel gas using steam, CO_2_, air, or O_2_ as gasifying agents.^6,7^ This fuel gas produced is usually a mixture of carbon dioxide (CO_2_), hydrogen (H_2_), carbon monoxide (CO), and methane (CH_4_). Synthesis gas (H_2_ + CO) is an important intermediate in the production of chemicals (ammonia, H_2_, or methanol) and biofuels; therefore, it is important to achieve the highest possible syngas yield during the gasification process.

Conventional gasification processes have the disadvantage of obtaining a fuel gas with a lower energy density as a result of its dilution in N_2_ when air is used as the gasifying agent and a higher economic penalty when pure O_2_ is used as the gasifying agent as a result of the need for an air separation unit (ASU). In addition, these processes lead to high tar generation, which means lower carbon conversion and increased risk of operational problems. Faced with this problem, dual fluidized bed (DFB) and chemical looping technologies, both based on interconnected fluidized bed reactors, have been studied as a possible solution.^8,9^ The DFB process has been studied on a large scale for biomass gasification with a capacity of 5 to more than 100 MW.^10^ It has the disadvantage that the heat required for gasification reactions is generated by burning char in the combustion reactor; therefore, the process generates significant CO_2_ emissions into the atmosphere.

**Figure 1:**
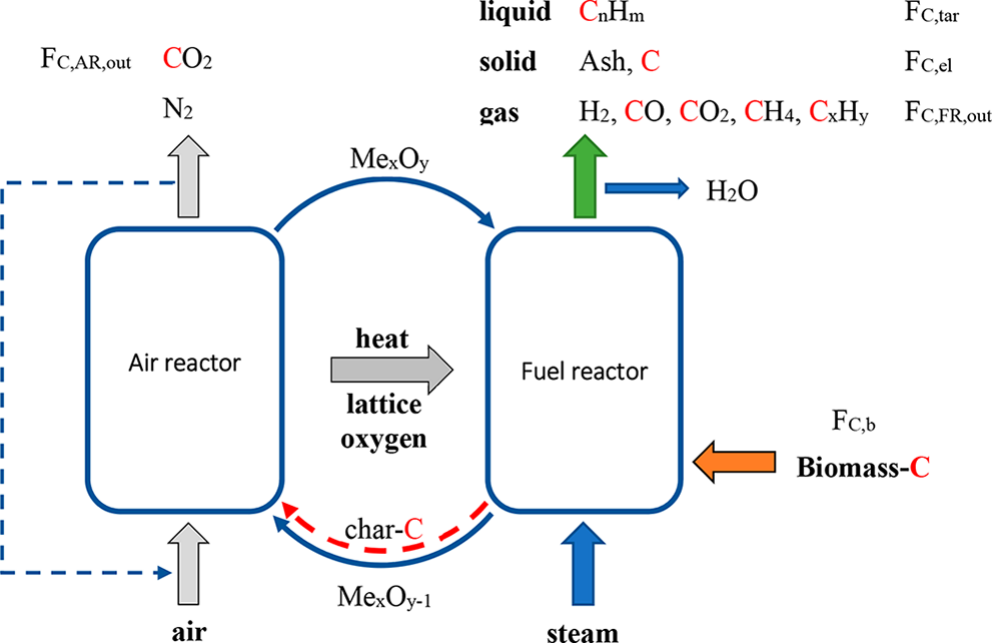
BCLG process diagram.

Biomass chemical looping gasification (BCLG) is an innovative process representing an alternative to conventional processes, allowing for higher efficiencies to be achieved at a lower operating cost.^11^ As shown in the schematic illustration of Figure 1, it is based on two interconnected fluidized bed reactors, where the bed material is a metal oxide (Me_*x*_O_*y*_) that is responsible for transporting lattice oxygen (less than the stoichiometric required for full combustion) and heat from the air reactor (AR) to the fuel or gasification reactor (FR), avoiding the mixture of gases between the reactors.^12^ The biomass is fed to the FR, where it decomposes, releasing volatile compounds (pyrolysis gas), tars, and char, which react with the gasifying agent (H_2_O or CO_2_) and the oxygen carrier, generating a mixture of CO_2_, H_2_, CO, CH_4_, and C_2_–C_3_. In these reactions, the oxygen carrier is reduced (Me_*x*_O_*y*–1_). The reduced oxygen carrier passes to the AR and is regenerated by oxidation with air to continue with a new cycle. In addition, the combustion of the char that can be drawn along with the oxygen carrier to the AR also occurs. The heat generated in the AR by the exothermic reactions is transported by the oxygen carrier to the FR to supply energy to the endothermic reactions.

The main advantages of BCLG with respect to conventional processes are that a high-quality syngas is obtained without diluting in N_2_ and without the need for an air separation unit. Furthermore, this process has low CO_2_ emissions to the atmosphere, with the potential to achieve net-negative emissions if combined with CO_2_ capture and storage technology,^13^ and allows for the reduction of the tar in the syngas because the oxygen carrier acts as a catalyst in the cracking of tars.^14^

The chemical looping combustion (CLC) process has reached high technological development during the past decade, and currently, it is considered among the lowest cost technologies for energy production with inherent CO_2_ capture.^15^ The chemical looping gasification (CLG) process is based on the same principles as CLC technology, although its objective is to obtain synthesis gas instead of heat, but its level of development is much lower. The BCLG technology has been tested in continuous operating units from 1.5 to 25 kW_th_.^9,12,16−22^

In both CLC and CLG, a key aspect of the process is the selection of the oxygen carrier. There has been extensive experience on the behavior of a wide variety of materials, mainly based on Fe, Mn, Ni, and Cu metal oxides, which have the capacity to transport lattice oxygen, although most of these materials have been studied in the CLC process.^15,23^

Natural and synthetic solid oxygen carriers have been proposed for the CLG process. The former is cheap, and the latter is more expensive, as a result of the preparation cost, but tends to perform better. The most studied synthetic materials for the BCLG process are based on Fe supported on an inert high-temperature material that contributes to increasing the reactivity and durability of the oxygen carrier. Although materials based on Fe as the only active phase have been studied,^16,18,20,24−26^ the use of bimetallic compounds based on mixtures of Fe, Ni, Cu, and Ba has also been analyzed to improve the efficiency of the process.^19,27−30^ However, most of these materials have only been tested in batch reactors. Working in continuous units, synthetic Fe_2_O_3_/Al_2_O_3_ oxygen carriers were used by Huseyin et al.^20^ and Wei et al.^18^ in a 10 kW_th_ unit and Sampron et al.^16^ in a 1.5 kW_th_ unit, to assess the behavior of the BCLG process using pine sawdust as fuel. Wei et al.^19^ also used a bimetallic Fe_2_O_3_/NiO oxygen carrier in their 10 kW_th_ unit. Finally, Ge et al.^21,22^ evaluated the behavior of different oxygen carriers based on Ni in a 25 kW_th_ CLG unit using rice husk as fuel.

From an economic perspective, natural oxygen carriers are more attractive as a result of their low cost. Fe-based minerals,^17,22,31^ Mn-based minerals,^32−34^ and even industrial waste, with a considerable content of transition metals, such as Linz–Donawitz (LD) slag,^35^ have been proposed for this process. Ge et al.^22^ used natural hematite mixed with silica sand to evaluate the gasification of rice husk in a 25 kW_th_ BCLG unit. Our research group analyzed the process behavior using ilmenite as the oxygen carrier and pine wood as fuel in a 1.5 kW_th_ unit. As far as we know, no studies with Mn-based ores in continuous units have been conducted.

In the experimental work performed in continuous units, it was observed that the most important parameter that affected the quality of the synthesis gas was the lattice oxygen transported from the AR to the FR. This parameter was varied through different options: (a) changing the biomass feeding rate and keeping the oxygen carrier circulation flow constant,^18,19^ (b) keeping the biomass feeding rate constant and varying the oxygen carrier circulation flow (the total solids circulating flow was kept constant, and the oxygen carrier/silica sand ratio was varied),^21,22^ and (c) in the case of our research group,^12,16^ keeping constant the biomass feeding rate and the oxygen carrier circulating flow and modifying the amount of oxygen fed to the AR. This method allowed us to vary this parameter, keeping the rest of the operating variables constant. Thus, it was possible to analyze the isolated effect of each process variable, such as the FR temperature and steam/biomass and oxygen/biomass ratios, without modifying the rest of the parameters.

This work aimed to study and compare the behavior of two natural ores, from Tierga (Spain) and Gabon, based on iron and manganese, respectively, during continuous operation in a 1.5 kW_th_ BCLG unit, with the effect of the main operating variables (FR temperature and oxygen/biomass and steam/biomass ratios) on the quality of the synthesis gas and the efficiency of the process. The comparison of the minerals was performed under similar operating conditions to attribute the differences in the process performance to the oxygen carrier used, avoiding the influence of other variables. Special emphasis has been considered in the determination of the tar content under steady-state operation at different operating conditions for both oxygen carriers.

## 2 Experimental Section

### 2.1 Materials

Two minerals were used as oxygen carriers in this work: Tierga iron ore from a hematite mine in Zaragoza (Spain) provided by PROMINDSA and manganese ore from Gabon provided by Ferroatlantica del Cinca S.L. Iron ore has been previously studied in the CLC process,^36^ while less data about manganese ore performance in chemical looping processes are available.

The raw materials were prepared by grinding and sieving to a particle size of 100–300 μm. They were calcined in an air atmosphere for 2 h at 950 °C for the iron ore and 800 °C for the manganese ore to increase the mechanical strength of the particles. Table 1 shows the main properties for the calcined ores used as oxygen carriers in this investigation.

**Table 1:** Physical Properties of the Calcined Ores Used as Oxygen Carriers

	Tierga Fe ore	Gabon Mn ore
XRD main phases^a^	76.5% Fe_2_O_3_, SiO_2_, Al_2_O_3_, CaO, and MgO	8.4% Fe_2_O_3_, 67.5% Mn_3_O_4_, and SiO_2_
particle diameter (μm)	100–300	100–300
skeletal density (kg/m^3^)	4216	3997
crushing strength (N)	5.8	1.8
porosity (%)	26.3	35.7

a Quantified by TGA.^36,37^

The crystalline phases in their oxidized oxygen carrier forms (Fe_2_O_3_, Fe_3_O_4_, FeO, Mn_3_O_4_, MnO, etc.) were analyzed qualitatively by means of Bruker D8 Advance crystalline powder X-ray diffraction (XRD). The content of the active compounds was determined by thermogravimetric analysis (TGA).^36,37^ Calcined samples showed that the iron ore contained 76.5 wt % Fe_2_O_3_ and manganese ore contained 67.5 wt % Mn_3_O_4_ and a small fraction of Fe_2_O_3_ and SiO_2_ phases.

The determination of the main elements present in the ores was carried out using an inductively coupled plasma optical emission spectroscopy (ICP–OES) Xpectroblue-EOP-TI FMT26 (Spectro) spectrophotometer working according to the corresponding ISO, ASTM, and UNE standards. Table 2 shows the results of the spectrophotometry analysis.

**Table 2:** Elemental Composition (wt %) of the Fresh Ores Determined by ICP–OES Spectrophotometry

	Tierga Fe ore	Gabon Mn ore
Al	2.1	3.8
Ca	3.8	
Fe	55	5.1
Mn	0.05	46.6
Si	3.8	4.2

The surface morphology of the samples was analyzed on a scanning electron microscopy–energy-dispersive X-ray analysis (SEM-EDX) Hitachi S-3400N variable pressure microscope up to 270 Pa with the EDX Röntec XFlash Si (Li) analyzer. The dispersion of the different compounds (Fe, Mn, etc.) along a section of the particle was also analyzed. Mercury porosimetry measurements were carried out on Micromeritics AUTOPORE V equipment according to ISO 15901 (1-2-3). TGA (TGA CI Electronics) using a mixture of 15 vol % H_2_ + 20 vol % H_2_O as the reducing agent and air for oxidation was performed to determine the evolution of reactivity and oxygen transport capacity of the materials during the operation.^38^ Moreover, 15 vol % CH_4_ + 20 vol % H_2_O was used to simulate reactions of volatile matter with oxygen carriers. The analysis revealed that the oxygen transport capacity (*R*_oc_) of fresh iron and manganese ores was 7.7 and 5.6 wt %, respectively, at 950 °C. This was the result of redox reactions involving the redox pair magnetite/wustite (Fe_2_O_3_/FeO) and hausmanite/manganosite (Mn_3_O_4_/MnO).

As a renewable solid fuel, industrial wood pellets (IWPs) from Darmstadt (Germany) were used. Table 3 shows the proximate and ultimate analyses of the biomass. The biomass particle size fed to the BCLG unit was 0.5–2 mm; therefore, it was necessary to grind and sieve the raw industrial wood pellets.

**Table 3:** Main Characteristics of the IWP Biomass

	IWP
Proximate Analysis (wt %, As Received)	
moisture	5.6
ash	0.6
volatile matter	78.5
fixed carbon	15.3
Ultimate Analysis (wt %, Dry Basis)	
C	52.6
H	6.0
N	0.1
S	0.0
O (by difference)	40.7
LHV (kJ/kg of dry biomass), EN 14918	18594
Ω_b_ (mol of O/kg of dry biomass)	92.5

### 2.2 1.5 kW_th_ Chemical Looping Gasification Unit at ICB–CSIC

**Figure 2:**
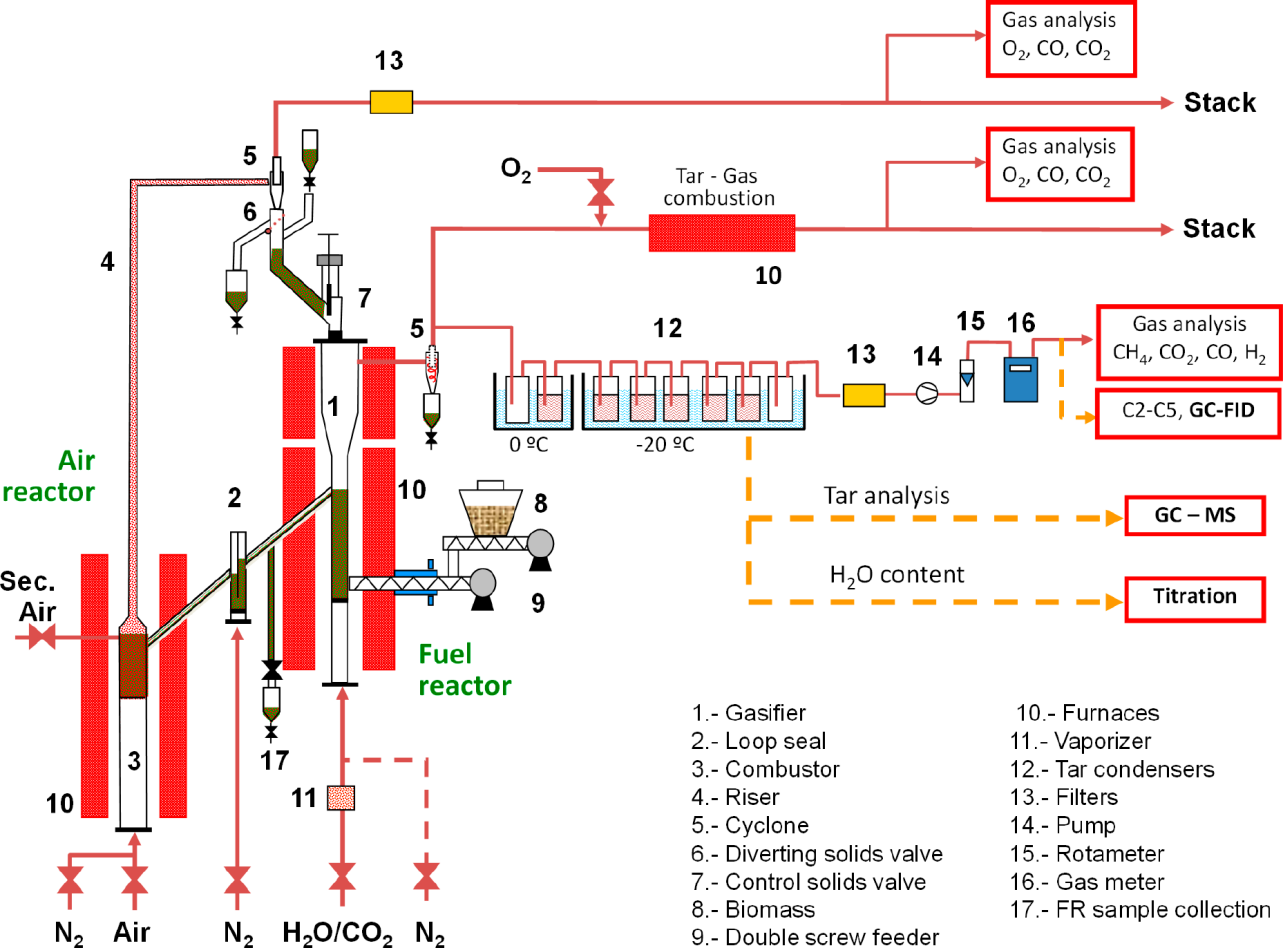
Scheme of the 1.5 kW_th_ CLG unit at ICB–CSIC.

The gasification tests were performed in a 1.5 kW_th_ chemical looping unit located at ICB–CSIC (Spain). Figure 2 shows the diagram of the unit. This unit consists of two fluidized beds, fuel and air reactors, interconnected by one loop seal that allows for the solid circulation and avoids the mixture of gases between both reactors. Furthermore, a solid circulation control valve is located at the upper entrance to the fuel reactor. This unit has a fuel supply system consisting of two screw feeders in series. The first screw feeder is connected to the biomass hopper and regulates the feeding rate by modifying the rotation speed of the screw. The second screw feeder is connected to the fuel reactor, and its function is to prevent the pyrolysis of biomass in the feeding pipe operating a high rotation speed.

The oxygen carrier is oxidized in the AR and in the riser with a stream of air diluted in N_2_. Solids are separated by a cyclone and returned to the FR through a solid valve that allows for control of the circulation rate. In the FR, the oxygen carrier is reduced when it reacts with the products of biomass gasification and circulates through the fluidized bed loop seal to the AR to start a new cycle. The inventory of solids in the unit was about 2.3 kg for the iron ore and 2.6 kg for the manganese ore.

In the AR, the solids were fluidized with an air/N_2_ mixture (2100 NL/h), thus controlling the oxygen supplied to the FR. The loop seal was fluidized with N_2_ (90 NL/h). Finally, steam or a mixture of steam and N_2_ (a total flow of 130 NL/h) was used as the fluidizing media in the FR. The steam flow rate was controlled by a water peristaltic pump that regulates the flow of water entering a vaporizer that operates at 150 °C. The other gases were fed by mass flow controllers. A more detailed description of the unit and the control of oxygen transferred to the FR was shown by Condori et al.^12^ and Samprón et al.^16^

The gas outlet streams from both reactors were continuously analyzed during the process. As seen in the diagram, the FR outflow was divided into two streams. One of them went to a post-combustor, where all of the gas was burned with oxygen and whose composition was subsequently analyzed (Siemens Ultramat/Oxymat 6 for CO_2_, CO, and O_2_). On the basis of the oxygen consumption and the composition of the post-combustor outlet, a better adjustment of the mass balances was made. The other part of the stream, at the outlet of the FR, was sent to a tar collection system that followed the European Tar Protocol.^39^ The water and tar contents of the recovered tar samples were analyzed by titration (Mitsubishi Karl Fischer titrator KF-31) and gas chromatography (GC-2010 with a Shimadzu QP2020 mass detector), respectively. This system cleaned the gas before reaching a paramagnetic O_2_ analyzer (Siemens Oxymat 6) and a non-dispersive infrared (NDIR) analyzer for CH_4_, CO_2_, and CO measurements and a thermal conductivity analyzer for H_2_ determination (SICK MAIHAK S710). Moreover, samples of this clean gas were collected and then analyzed in a gas chromatograph (CLARUS 580 PerkinElmer) to know the light hydrocarbon content (C_1_–C_5_).

For the analysis of the AR output stream, two analyzers (Siemens Ultramat 23 and Oxymat 6) were used to determine the concentration of the gases CO_2_, CO, and O_2_.

### 2.3 BCLG Reactions with Oxygen Carriers

Gasification reactions have been widely studied for different types of fuels (coal, biomass, etc.). When the fuel (biomass) is fed to the FR, it is dried and pyrolyzed/devolatilized, generating gases (mainly hydrogen, carbon monoxide, carbon dioxide, methane, and light hydrocarbons), tars, and char (reaction R1). This is followed by a large number of simultaneous reactions among the different pyrolysis products, the gasifying agent (steam or CO_2_), and the solid oxygen carrier, which are shown below.

The char is gasified with steam (reaction R2) or CO_2_ (reaction R3) generating CO + H_2_ or CO, respectively. Simultaneously, redox reactions take place between the active phases of the oxygen carrier (Me_*x*_O_*y*_) and the gases generated during the biomass pyrolysis and the char gasification (reactions R4–R7). Methane and light hydrocarbons are reformed with steam or CO_2_ to give synthesis gas (reactions R8 and R9). Finally, the water–gas shift (WGS) reaction also takes place (reaction R10). The tar removal reactions deserve special attention. Shen et al.^40^ compiled the major reactions occurring involving tar, which mainly depend upon the temperature or the presence of a catalyst. These reactions include thermal cracking (reaction R11), hydrocracking/dealkylation (reaction R12), dry reforming, and steam reforming/dealkylation (reaction R13). In addition, reactions between the oxygen carrier and tar also appear during the BCLG process (reaction R14).
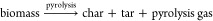
R1
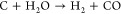
R2
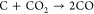
R3
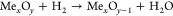
R4
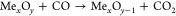
R5
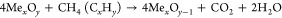
R6
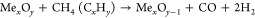
R7
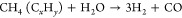
R8
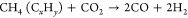
R9
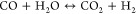
R10
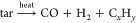
R11
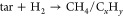
R12
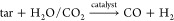
R13
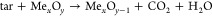
R14Finally, the oxygen carrier reduced in the FR (Me_*x*_O_*y*–1_) reaches the AR, where it is oxidized with air (reaction R15), being prepared to start a new redox cycle. Another reaction that can occur in the AR is the combustion of the char transferred together with the oxygen carrier from the FR (reaction R16).
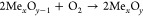
R15
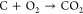
R16In all reactions involving iron and manganese ores, Me_*x*_O_*y*_ is Fe_2_O_3_ or Mn_3_O_4_ and Me_*x*_O_*y*–1_ is FeO or MnO.

### 2.4 Operating Parameters and Data Evaluation

Previous investigations indicated that the main operating variables that affected the BCLG process behavior were the oxygen/biomass ratio, λ, the fuel reactor temperature, and the steam/biomass ratio, S/B.^12^

The oxygen/biomass ratio, λ, represents the amount of oxygen transferred to the FR by the oxygen carrier. It is calculated as the ratio between oxygen fed to the AR and the stoichiometric oxygen necessary for the complete combustion of the biomass.
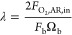
1All oxygen fed to the AR reacts there (pure N_2_ is obtained at the outlet of the AR). Oxygen reacted with the oxygen carrier is transferred to the FR as lattice oxygen to produce synthesis gas.

The total oxygen demand is calculated from the ultimate biomass analysis using the following equation:
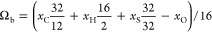
2The steam/biomass ratio, S/B, is the relationship between the amount of water fed into the fuel reactor, including biomass moisture, and the amount of dry biomass fed into the fuel reactor.

The parameters used to evaluate the performance of the BCLG process were the following:

Biomass conversion, *X*_b_, is defined as the fraction of carbon converted to gas in the fuel and air reactors with respect to the total carbon fed to the unit.
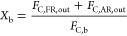
3
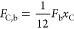
4
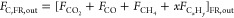
5
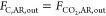
6

Carbon conversion efficiency, η_cc_, is the fraction of carbon converted to gas in the fuel reactor with respect to total carbon converted to gas in the whole system.
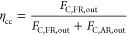
7

Syngas yield, *Y* (Nm^3^/kg of dry biomass), considers the amount of H_2_ and CO produced with respect to the dry biomass fed to the unit.
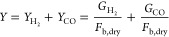
8

The H_2_/CO ratio considers the ratio between the production of H_2_ and CO in the process. It depends upon the gasifying agent used, and it is useful to consider the final use of the syngas.
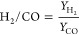
9

Cold gas efficiency, η_g_, is the fraction of chemical energy contained in the product gas from the fuel reactor over the total energy of the biomass.
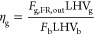
10

## 3 Results

The comparison of the performance of iron and manganese ores, as oxygen carriers for the BCLG process, was carried out by investigating the effect of the main operating variables (fuel reactor temperature and oxygen/biomass and steam/biomass ratios) on biomass conversion, carbon conversion efficiency, syngas yield, cold gas efficiency, and tar generation. A complete study of both materials was performed in the 1.5 kW_th_ CLG unit during 104 h of operation under high-temperature conditions, of which 88 h were under gasification conditions feeding biomass (40 h for Tierga Fe ore and 48 h for Gabon Mn ore). The investigation of the effect of each variable was carried out keeping the rest of the operating conditions constant and close to the autothermal conditions (*T* = 930–950 °C, S/B = 0.6, and λ = 0.3–0.4) for each oxygen carrier. The control of the oxygen fed to the AR, by diluting the air with N_2_, allowed for the study of the effect of the oxygen/biomass ratio, λ, in the CLG process. For the study of the steam/biomass ratio, S/B, the amount of steam introduced to the FR was modified, replacing it with N_2_ but keeping the total flow constant and, therefore, the same fluidization velocity in the FR in all cases.

The solid inventories required for filling the pilot plant with iron and manganese ores were 2.3 and 2.6 kg, respectively. Both materials were loaded and circulated at 900 °C in air atmosphere for 8 and 5 h, respectively, to remove fine particles that were not removed during the sieving because they remain stuck to large particles. After that, biomass was fed and the fluidization gases were changed to set the CLG conditions (steam as the gasification agent for the FR and air/N_2_ mixture for the AR). At the end of each experimental day, the unit was cooled in a nitrogen atmosphere to maintain the oxygen carrier in the reduced form and prevent its oxidation. In all tests, IWPs (from Darmstadt, Germany) were used as fuel.

Each test involved at least 2 h of operation. More than 1 h was necessary to ensure that the steady state has been reached, and during the following hour, a continuous analysis of the flue gas composition was carried out maintaining steady-state conditions. Additional time was spent in those tests where tar measurements were carried out. Table 4 shows the experimental operating conditions used and results obtained as mean values, which describe the behavior of the BCLG process under different operating conditions for the iron and manganese ores. The standard deviation for each experimental data during steady-state operation is included in the table.

**Table 4:** Test Data and Results from 1.5 kW_th_ BCLG Continuous Operation Using “Tierga” Iron and “Gabon” Manganese Ores as the Oxygen Carrier and IWPs as Fuel, with Power at 1 kW

				gas composition (dry and N_2_ free, vol %)									
test	*T*_FR_ (°C)	S/B kg/kg	λ	CO_2_	CO	H_2_	CH_4_	C_2_–C_3_	*X*_b_ (%)	η_cc_ (%)	η_g_ (%)	*Y* (Nm^3^/kg)	H_2_/CO
Fe Ore													
1	817	0.61	0.31	36.7 ± 1.6	21.3 ± 0.6	29.2 ± 0.7	10.7 ± 0.2	2.12 ± 0.1	90.0 ± 0.1	88.8 ± 0.4	64.8 ± 2.4	0.55 ± 0.02	1.37 ± 0.07
2	818	0.62	0.42	47.5 ± 3.2	16.6 ± 0.9	25.4 ± 1.9	8.9 ± 0.1	1.67 ± 0.1	98.4 ± 1.2	89.4 ± 0.2	56.6 ± 3.6	0.48 ± 0.04	1.53 ± 0.20
3	825	0.57	0.51	53.6 ± 0.8	16.2 ± 0.2	18.0 ± 0.3	9.5 ± 0.2	2.66 ± 0.1	98.1 ± 0.1	90.0 ± 0.2	51.3 ± 1.2	0.36 ± 0.01	1.11 ± 0.03
4	885	0.63	0.31	42.5 ± 0.5	18.6 ± 0.2	27.0 ± 0.2	10.5 ± 0.1	1.41 ± 0.1	92.7 ± 2.7	98.3 ± 0.1	67.2 ± 1.3	0.57 ± 0.01	1.45 ± 0.05
5	870	0.64	0.42	49.9 ± 2.6	16.1 ± 1.7	22.4 ± 1.5	9.6 ± 0.3	1.98 ± 0.1	95.4 ± 1.5	95.6 ± 0.1	56.7 ± 3.0	0.44 ± 0.03	1.39 ± 0.27
6	947	0.05	0.41	45.0 ± 3.3	26.9 ± 1.5	16.8 ± 1.1	8.6 ± 0.4	2.66 ± 0.2	87.7 ± 0.8	96.2 ± 3.1	54.8 ± 4.7	0.44 ± 0.04	0.63 ± 0.07
7	944	0.36	0.41	45.7 ± 2.0	21.5 ± 0.6	20.3 ± 0.7	9.7 ± 0.4	2.87 ± 0.1	88.5 ± 0.4	98.8 ± 0.2	58.0 ± 3.0	0.44 ± 0.02	0.95 ± 0.05
8	947	0.62	0.21	30.8 ± 2.6	24.7 ± 0.8	33.5 ± 1.6	8.7 ± 0.1	2.30 ± 0.2	93.7 ± 1.0	99.5 ± 0.5	83.2 ± 3.9	0.79 ± 0.04	1.35 ± 0.12
9	940	0.58	0.31	41.3 ± 4.8	20.7 ± 1.4	26.5 ± 2.8	9.7 ± 0.3	1.85 ± 0.2	93.6 ± 0.8	99.5 ± 0.5	67.9 ± 7.0	0.59 ± 0.06	1.28 ± 0.24
10	935	0.62	0.41	49.3 ± 1.0	17.2 ± 0.4	21.7 ± 0.3	9.9 ± 0.2	1.80 ± 0.1	92.1 ± 0.3	98.0 ± 0.1	56.1 ± 1.8	0.44 ± 0.01	1.26 ± 0.05
11	938	0.62	0.52	55.9 ± 0.6	16.2 ± 0.3	17.5 ± 0.2	8.8 ± 0.1	1.60 ± 0.1	97.8 ± 0.4	98.9 ± 0.1	50.4 ± 1.1	0.39 ± 0.01	1.08 ± 0.03
Mn Ore													
1	818	0.66	0.20	27.7 ± 1.1	22.7 ± 0.5	37.8 ± 0.4	9.4 ± 0.1	2.33 ± 0.2	82.2 ± 1.1	87.6 ± 0.1	71.1 ± 2.7	0.68 ± 0.02	1.66 ± 0.06
2	820	0.66	0.31	34.1 ± 1.5	21.7 ± 0.3	32.0 ± 0.8	9.5 ± 0.2	2.77 ± 0.1	86.8 ± 0.2	87.2 ± 0.1	65.4 ± 2.4	0.57 ± 0.02	1.47 ± 0.06
3	817	0.59	0.40	47.3 ± 1.1	17.4 ± 0.4	23.5 ± 0.6	9.2 ± 0.1	2.51 ± 0.1	92.3 ± 0.8	90.4 ± 0.1	54.9 ± 2.0	0.43 ± 0.02	1.35 ± 0.07
4	870	0.60	0.30	35.7 ± 1.6	21.7 ± 0.5	31.8 ± 0.9	9.0 ± 0.2	1.73 ± 0.1	94.6 ± 0.3	92.6 ± 0.1	71.1 ± 2.4	0.67 ± 0.02	1.46 ± 0.08
5	877	0.59	0.40	46.8 ± 2.1	19.0 ± 0.7	23.8 ± 1.1	8.7 ± 0.1	1.68 ± 0.2	93.4 ± 0.6	94.8 ± 0.1	56.2 ± 3.4	0.49 ± 0.03	1.25 ± 0.11
6	922	0.05	0.30	22.1 ± 2.0	39.4 ± 0.9	29.0 ± 0.7	8.6 ± 0.1	0.87 ± 0.3	90.2 ± 1.1	92.0 ± 0.1	71.5 ± 5.7	0.76 ± 0.06	0.73 ± 0.04
7	926	0.31	0.30	31.0 ± 1.1	29.6 ± 0.5	30.5 ± 0.4	8.1 ± 0.1	0.81 ± 0.1	94.4 ± 0.7	95.7 ± 0.1	70.4 ± 4.4	0.75 ± 0.04	1.03 ± 0.03
8	923	0.59	0.21	27.7 ± 0.8	25.5 ± 0.3	37.6 ± 0.3	8.1 ± 0.1	0.99 ± 0.1	90.3 ± 0.4	95.8 ± 0.1	79.7 ± 4.3	0.86 ± 0.02	1.47 ± 0.03
9	925	0.59	0.31	37.8 ± 1.6	22.2 ± 0.9	30.8 ± 1.0	8.7 ± 0.3	1.67 ± 0.1	95.1 ± 0.1	96.5 ± 0.1	73.6 ± 1.3	0.70 ± 0.02	1.39 ± 0.1
10	926	0.64	0.41	46.2 ± 0.7	20.2 ± 2.3	23.1 ± 0.8	9.2 ± 0.1	1.26 ± 0.1	93.4 ± 2.7	96.4 ± 0.1	56.6 ± 1.9	0.50 ± 0.01	1.14 ± 0.2

### 3.1 Effect of the Oxygen/Biomass Ratio, λ

The oxygen/biomass ratio represents the lattice oxygen that is transported by the oxygen carrier from the AR to the FR for the gasification/combustion of the biomass. This was the most relevant operating variable because it directly affects the quality of the syngas produced.^41^ Furthermore, the λ value is also very important to achieve the autothermal operating conditions of the system. In this work, this parameter was varied by controlling the oxygen fed to the AR, by diluting of the air with N_2_.

**Figure 3:**
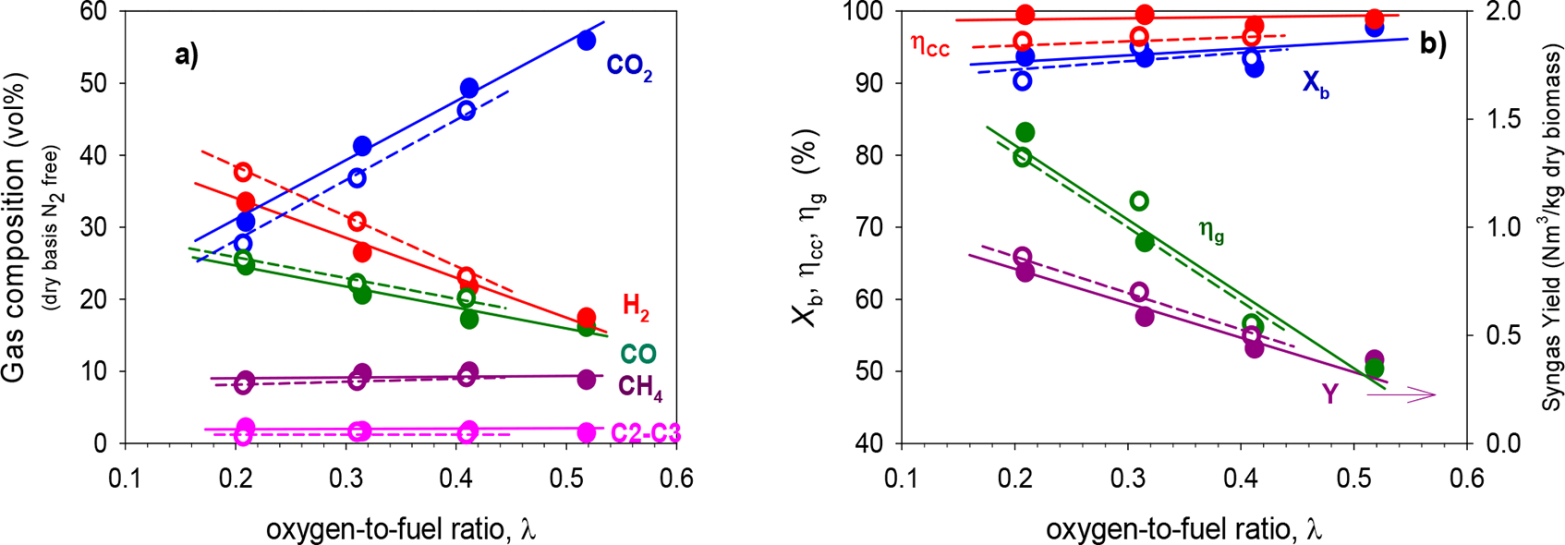
Effect of the oxygen/biomass ratio, λ on (a) gas composition (dry and N_2_ free) and (b) biomass conversion, *X*_b_, carbon conversion efficiency, η_cc_, cold gas efficiency, η_g_, and syngas yield, *Y*. *T* ≈ 940 °C, and S/B ≈ 0.6. Iron ore (full dots, continuous lines) and manganese ore (empty dots, dashed lines).

Figure 3 shows the effect of the oxygen/biomass ratio on the syngas composition, process efficiencies, and syngas yield at 940 °C and S/B ratio of about 0.6 using both iron and manganese ores.

It can be observed that, with both oxygen carriers, the CO_2_ concentration increased with increasing λ values because more lattice oxygen was transported toward the FR, promoting the combustion reactions of the gases generated by gasification and pyrolysis (reactions R1–R3). Furthermore, lower λ values, corresponding to lower lattice oxygen transport, led to the production of higher quality syngas, with higher CO and H_2_ concentrations.

The oxygen carrier is reduced in the FR by the gases generated during biomass devolatization and gasification and is oxidized in the AR with oxygen through exothermic reactions. The energy necessary for endothermic reactions that occurs in the FR is supplied by the hot oxygen carrier coming from the AR, avoiding the need for an external energy input. According to the calculations carried out by Sampron et al.,^41^ λ values between 0.33 and 0.38 are required to operate under autothermal conditions in the BCLG process.

On the other hand, it was observed that the amount of CH_4_ and light hydrocarbons (C_2_–C_3_) was high (around 10%), and surprisingly, this was hardly affected by the amount of lattice oxygen transported to the FR. This fact means that the reactions between these gases, coming from biomass devolatilization, and the oxygen carrier are not favored as a result of a low reaction rate and contact time in the fluidized bed. Other authors have found similar results using other Fe-based oxygen carriers.^12,16,18,22^

The gas composition obtained with the two ores was similar for the same operating conditions, and both exhibited a similar trend with respect to the variation of λ. It can be seen that slightly higher CO_2_ concentrations were achieved with iron ore and, as a result, slightly lower H_2_ and CO concentrations. However, the amount of CH_4_ and C_2_–C_3_ obtained with both minerals was quite similar.

From the analysis of the process efficiency, the variation of λ had an important impact on the syngas yield (*Y*) and the cold gas efficiency (η_g_). These parameters were greatly affected because they depended directly upon the composition of the syngas produced. An increase in the value of λ led to a lower syngas yield and cold gas efficiency because syngas was partially consumed in the combustion reactions. Manganese ore from Gabon generated slightly higher syngas yields than Tierga iron ore because it produced slightly higher H_2_ and CO concentrations. On the contrary, biomass conversion (*X*_b_) and carbon conversion efficiency (η_cc_) were hardly affected by the variation of λ. In the best conditions (high temperature and S/B ratios of around 0.6), biomass conversion and carbon conversion efficiency were always above 90 and 96%, respectively, using both ores. It should be noted that iron ore achieved higher carbon conversion efficiencies but with very small differences compared to manganese ore.

In comparison of the results obtained in this work to those obtained by other researchers, it was observed that the main difference with respect to the effect of λ was in the carbon conversion efficiency. In this work, the value of λ hardly affected the carbon conversion efficiency; that is, it hardly affected the CO_2_ emissions in the AR. However, other researchers observed an increase in carbon conversion efficiency with increasing λ.^18,22^ This difference may be attributed as a consequence of the different method used to control oxygen transferred to the FR. In the unit used in this work, oxygen fed to the AR reacted competitively with the oxygen carrier and with carbon coming from the FR. To decrease the λ value, oxygen fed to the AR was reduced. Therefore, the lower the oxygen concentration, the lower the amount of char burned. However, when the λ value decreased, the amount of oxygen transferred to the FR decreased, and as a consequence, the gasification rate of the char in this reactor was slower, its concentration increased, and more char passed to the AR. Both effects were offset, and carbon conversion efficiencies were practically unaffected by the λ value. It should be noted that, using this method to control oxygen, part of the char that passed to the AR was burned and part was recirculated with the oxygen carrier to the FR. Wei et al.^18^ investigated the effect of λ by varying the biomass feeding rate and keeping the oxygen carrier circulation rate constant. As the biomass feed rate increased, λ decreased, more carbon passed from the FR to the AR, where it was burned with the air fed to this reactor, and as a consequence, the carbon conversion efficiency decreased. Ge et al.^22^ varied the λ value by changing the mass percentage of hematite in the mixture of silica sand and natural hematite used as bed material. As the mass percentage of hematite in the mixture increased (higher λ), the carbon conversion efficiency increased and less CO_2_ was emitted in the AR.

### 3.2 Effect of the Gasification Temperature

The gasification temperature can be another important variable that affects the behavior of the BCLG process, because it affects the devolatilization process and the gasification rate of the char generated after devolatilization. In this work, the influence of the temperature in the FR, in the usual range of temperatures used in gasification processes (820–940 °C), on the efficiency of the process and the composition of the synthesis gas obtained was studied. For this, the rest of the operating variables were kept constant and close to the autothermal conditions (λ ≈ 0.3 and S/B ≈ 0.6).

**Figure 4:**
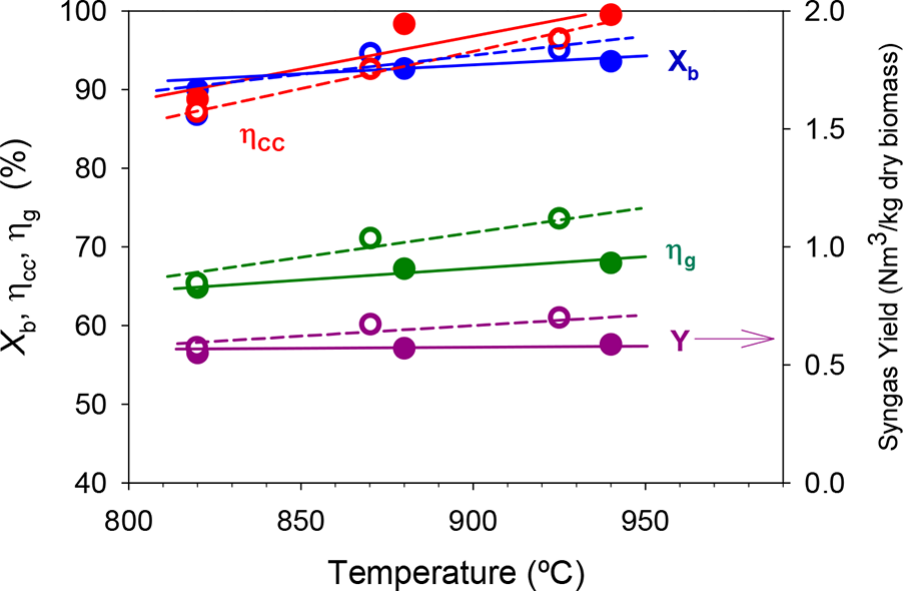
Effect of the fuel reactor temperature on biomass conversion, carbon conversion efficiency, cold gas efficiency, and syngas yield using Fe Tierga (full dots, continuous lines) and Mn Gabon ore (empty dots, dashed lines) as the oxygen carrier. λ = 0.3, and S/B = 0.6.

The experimental operating conditions used and the syngas composition obtained with the two ores are shown in Table 4. It was observed that the temperature did not have a significant impact on gas composition for any ore. Moreover, no differences in the concentration of hydrocarbons, CH_4_, and C_2_–C_3_ were observed with increasing the temperature. The CO_2_ concentration was always slightly lower and the H_2_ and CO concentrations were slightly higher for the manganese ore that for iron ore, which was reflected in slightly higher syngas yields, as shown in Figure 4. This figure also shows the effect of the temperature on the biomass conversion, carbon conversion efficiency, and cold gas efficiency. No significant differences were observed in the behavior of both ores.

The parameter most affected by the temperature was the carbon conversion efficiency, η_cc_, with values around 88% at the lowest temperature used, 820 °C, and reaching values higher than 96% with manganese ore and close to 100% with iron ore at the highest temperature used, 940 °C. This fact was due to the improvement of the biomass gasification in the FR when the temperature increased. The biomass conversion was little affected by the temperature in the case of iron ore, reaching a stable value of around 93% from 880 °C. However, with manganese ore, the conversion followed an increasing trend with the temperature reaching a value of around 95% at 930 °C. The syngas yield and cold gas efficiency increased a little with increasing temperature as a result of the higher carbon conversion produced in the FR, reaching values of around 0.6 Nm^3^/kg of dry biomass and 68%, respectively, at *T* ≈ 940 °C, λ ≈ 0.3, and S/B ≈ 0.6.

Wei et al.^18^ analyzed a wider temperature range (670–900 °C) than that used in this work and obtained very similar results to those obtained in this work, even using a different method to control the oxygen transferred from the AR to the FR. Ge et al.^22^ also observed that carbon conversion efficiency increased with increasing temperature. However, these researchers found that the optimum temperature for the BCLG process was 860 °C, because at this temperature, the maximum generation of syngas occurred. This maximum was the result of two opposite effects. As the temperature increased, the gasification rate increased and more biomass was converted to H_2_ and CO in the fuel reactor, increasing the production of syngas. However, as the temperature increased, the oxygen carrier oxidized more H_2_ and CO, reducing the concentration of these gases and reducing the generation of syngas. The greater oxidation of H_2_ and CO was a consequence of the greater amount of oxygen transported from the AR to the FR, because in the tests, it was observed that the oxygen concentration in the AR decreased slowly with increasing temperature. Therefore, in the tests carried out by Ge et al.,^22^ the increase in the temperature was associated with a small increase in the oxygen/biomass ratio as a result of the method used to control the oxygen transferred from the AR to the FR. That was the cause of the difference between the results found by these researchers and those observed in this work.

### 3.3 Effect of the Steam/Biomass Ratio, S/B

The steam/biomass ratio, S/B, is another important operating variable to be considered in gasification processes, because steam is commonly used as the gasifying agent and fluidization gas of the FR. Although a higher steam feed could improve the gasification and reforming reactions in the FR, its production also implies an energy penalty that could affect the heat balance of the whole process.

**Figure 5:**
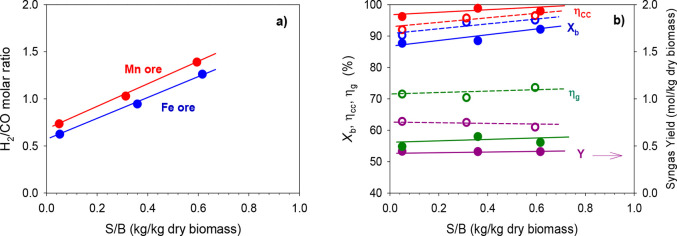
Effect of the S/B ratio on biomass conversion, carbon conversion efficiency, cold gas efficiency, syngas yield, and H_2_/CO ratio using Fe Tierga (full dots, continuous lines in b) and Mn Gabon ores (empty dots, dashed lines in b) as the oxygen carrier. *T* ≈ 940 °C, and λ ≈ 0.4 for iron ore and 0.3 for manganese ore.

The effect of the steam/biomass ratio, S/B, was investigated in the range of 0.05–0.65 at a gasification temperature in the FR of 930–940 °C and with an oxygen/biomass ratio close to autothermal. In all of the tests, a constant flow of gas was fed to the FR (130 NL/h of steam/N_2_) to maintain the fluidization of the solids in the process. The amount of water fed was varied, and the total flow was completed with N_2_. In the tests in which no steam was fed, all of the flow was N_2_, and the value of the S/B ratio was 0.05 as a result of the moisture of the biomass. The experimental operating conditions used and the syngas compositions obtained with the two ores are shown in Table 4. The variations of the process efficiency parameters, the syngas yield, and the H_2_/CO ratio with respect to the S/B ratio for both natural minerals are shown in Figure 5.

In general, with both ores (test 6-7-10 for Fe ore and test 6-7-9 for Mn ore), it was observed that an increase in the S/B ratio produced an increase in CO_2_ and H_2_ concentrations and a decrease in the CO concentration, which was more significant for the case of the Mn ore as a result of the lower λ used. This effect was mainly due to the equilibrium of the WGS reaction (reaction R10), which was shifted toward the production of CO_2_ and H_2_ in the presence of a greater amount of water, consuming CO and H_2_O. As a consequence of this variation in the gas composition, the most affected parameter for both ores was the H_2_/CO ratio, showing an increasing trend as the S/B ratio increased (see Figure 5). However, the reforming reaction rates of methane and light hydrocarbons (C_2_–C_3_) were hardly affected by the increase of the S/B ratio. The Fe ore reached concentrations close to 10% CH_4_ and 2.5% C_2_–C_3_, while the Mn ore reached concentrations around 8.6% CH_4_ and 1.5% C_2_–C_3_. Other studies also did not show a significant effect of the S/B ratio in reforming of methane and hydrocarbons.^22^

With regard to the parameters that show the efficiency of the process, it was observed that the increase in the S/B ratio produced a slight increase in the conversion of biomass, as a consequence of a higher gasification rate, reaching values between 88 and 92% for Fe ore and between 90 and 95% for Mn ore. The carbon conversion efficiency always reached values above 92%, following an upward trend to 96% for Mn ore. In the case of Fe ore, the carbon conversion efficiency was hardly affected, with values close to 100% (>97%). The small effect of the S/B ratio on these parameters was logically due to the high values obtained even in the most unfavorable conditions (low S/B ratios). Likewise, as a result of the low influence of the S/B ratio on biomass conversion and carbon conversion efficiency, the syngas yield and the cold gas efficiency were hardly affected. With iron ore, syngas yield and cold gas efficiency values of around 0.44 Nm^3^/kg of dry biomass and 55%, respectively, were obtained. With Mn ore, the syngas yield and the cold gas efficiency values obtained were around 0.7 Nm^3^/kg of dry biomass and 72%, respectively. This considerable difference between the two minerals was mainly due to the difference in λ used for the study of the isolated effect of the S/B ratio.

Ge et al.^22^ analyzed the effect of the S/B ratio in the range of 0.6–1.4 by changing the steam flow rate while keeping the biomass feeding rate constant and observed an increase in CO_2_ and H_2_ concentrations and a decrease in the CO concentration as the S/B ratio increased. However, these researchers found that the biomass conversion reached the maximum at the S/B of 1.0 and that the syngas yield increased first and then remained constant. The maximum biomass conversion at the S/B of 1.0 was explained as a result of two opposite effects. With the increase of the S/B ratio, the biomass gasification rate was promoted and, therefore, its conversion was improved, but the elutriation of fine biomass char particles from the FR was also increased, decreasing the conversion of the biomass. The first effect was predominant for S/B ratios of <1.0, and the second effect was predominant for S/B ratios of >1.0. However, as previously mentioned, in all of the tests carried out in this work, a constant flow of gas was fed to the FR to maintain the fluidization conditions constant in the process. Therefore, in our tests, there was no increase in char particle elutriation with increasing S/B ratio, and thus, the maximum in the biomass conversion was not observed.

### 3.4 Tar Content

The amount of tar generated during the biomass gasification process was also investigated. An issue in conventional gasification processes is the generation of a large amount of tar that must be removed during the syngas cleaning step, which is usually expensive. In addition, if the end use of the syngas is the production of synthetic liquid fuels (via Fischer–Tropsch) or chemicals, it is necessary to minimize both the tar generation in the gasifier and the operation problems.

Previous investigations have shown that the BCLG process represents a good option for syngas production with low tar generation, because the presence of an oxygen carrier in the system helps its reduction.^5,12,16^ It was observed that the operational variables with the greatest impact on the tar content were the fuel reactor temperature and the steam/biomass ratio.

The effect of the FR temperature was analyzed in the range of 820–940 °C, keeping the rest of the variables constant (λ = 0.3 and S/B = 0.6) for the two ores. The effect of the S/B ratio was also analyzed at 930 °C with values of λ = 0.4 for iron ore and λ = 0.3 for manganese ore.

**Figure 6:**
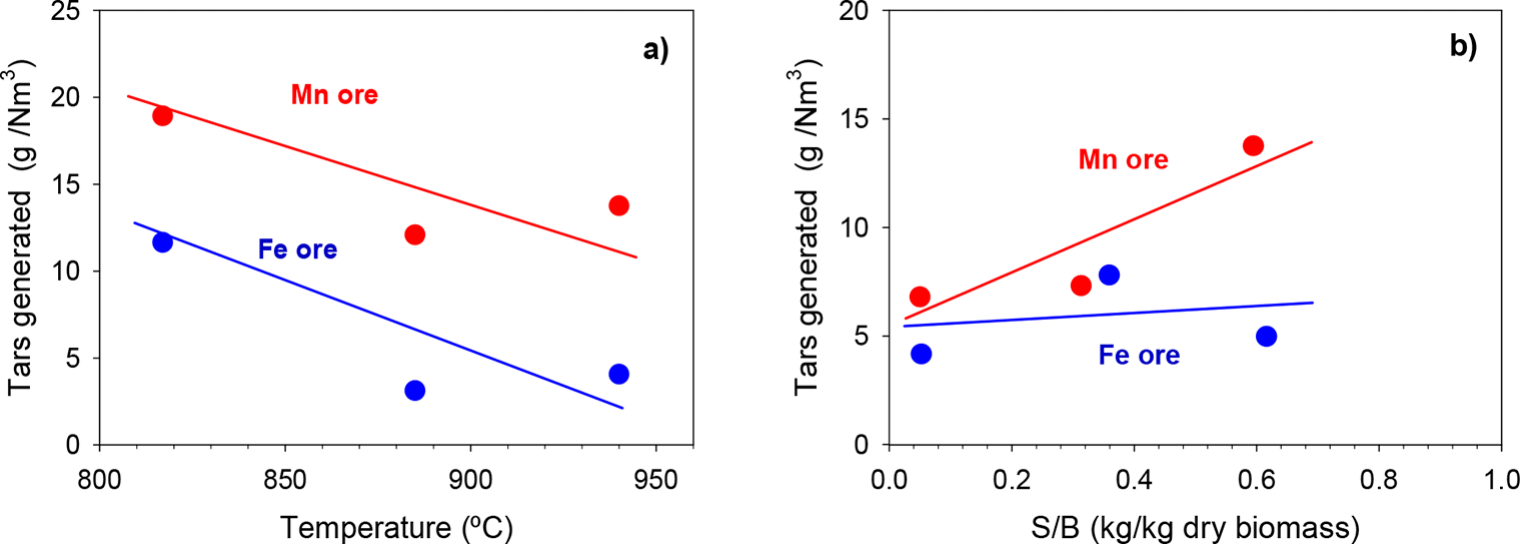
Tar generation during the BCLG process using Fe and Mn ores as the oxygen carrier: (a) effect of the FR temperature, with S/B = 0.6 and λ = 0.3, and (b) effect of the S/B ratio at *T* ≈ 940 °C, with λ = 0.4 for Fe ore and 0.3 for Mn ore.

For both materials, a lower amount of tar was generated at higher temperatures. Tar contents were always higher using the Mn ore as the oxygen carrier than using the Fe ore, being in the range of 3–12 and 12–19 g/Nm^3^ for the iron and manganese ores, respectively. This effect was due to the fact that an increase in the temperature led to an increase in the thermal cracking and combustion reactions of tars with the oxygen carrier. Figure 6 also shows the increase in the tar content with increasing the S/B ratio in a way similar to the results found by Virginie et al.^14^ It is well-known that iron in the elemental state has catalytic properties for the tar reforming reaction. However, the thermodynamic and kinetic limitations of the BCLG process usually do not allow for such a state to be achieved. Thus, this effect may be because some of the iron oxide was forced to reach a higher state of reduction when there was a lower concentration of water in the FR (lower S/B ratio) when the reduction potential of gases is higher. In the extreme case, where no water was introduced (S/B = 0.05), values of 4 and 7 g/Nm^3^ were reached for iron and manganese ores, respectively.

BCLG studies carried out with other oxygen carriers found the same decreasing tendency with the temperature, reaching values of around 1.5–3.7 g/Nm^3^ for ilmenite and 2.3–3.5 g/Nm^3^ for LD slag under similar conditions.^12,45^ In contrast, it was observed that an increase in the S/B ratio led to an increase in the amount of tars generated using ilmenite.

Anyway, in general, the values found for both ores (iron and manganese) were higher than those found with ilmenite. Only when iron ore was used under optimal conditions, the amount of tar generated was very similar to that observed with ilmenite. In addition, the use of iron ore under optimum conditions (high temperature, λ = 0.3–0.4, and S/B = 0.6) produced less tar amounts than those found in the indirect gasification process (DFBG) using silica sand (17 g/Nm^3^) and olivine (5.1 g/Nm^3^) as bed materials.^14^

### 3.5 Fluid Dynamic Behavior of Iron and Manganese Ores

A condition that an oxygen carrier must fulfill to be used as bed material in chemical looping processes is to have high attrition resistance, because it will increase its lifetime in the process. In contrast, a high attrition rate would increase the oxygen carrier makeup in the process and, therefore, the operating costs.

When operating in a circulating bed regime, the oxygen carrier is exposed to the friction between the particles and the collision against the reactor wall. Note that the CLG process subjects the oxygen carrier to more intense chemical stress than the CLC process because it is highly reduced in the fuel reactor; therefore, lower lifetime values are expected.^12^

**Figure 7:**
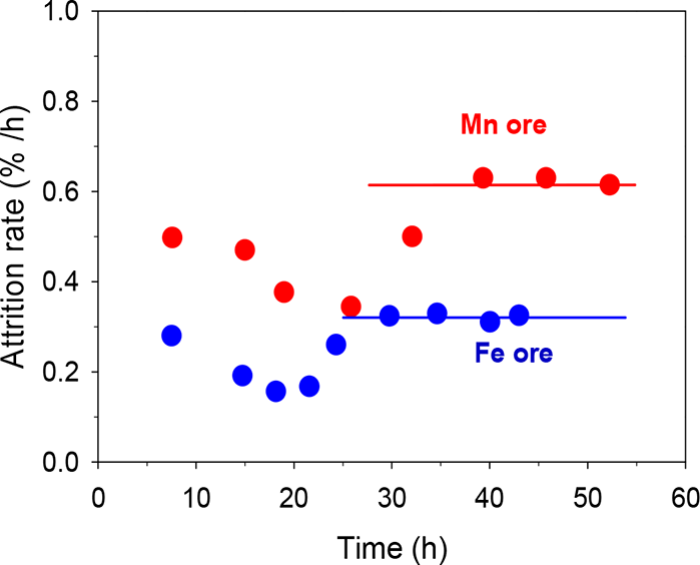
Comparison of the attrition rate of the Fe and Mn ores during BCLG continuous operation.

Attrition was investigated experimentally by collecting the fines released by the particles in cyclones located downstream of the AR and FR. The weight of fines with a size lower than 40 μm elutriated by the unit over the operating time under reducing conditions was quantified. Figure 7 shows the evolution of the amount of fines generated during the CLG process versus the operating time at a high temperature for both ores. As mentioned above, 40 and 48 h correspond to gasification conditions for the Fe and Mn ores, respectively.

At the beginning, the iron ore showed some operation difficulties as a result of the large amount of fines stuck to the particles in the fresh material that impeded a correct circulation between reactors. Once they were eliminated, the fluid dynamic behavior was good. The manganese ore did not show these initial problems, although the manganese ore showed a higher attrition rate compared to the iron ore, reaching stable values of 0.62 and 0.33%/h, respectively. These values correspond to a lifetime of 160 h for the Mn ore and 300 h for the Fe ore. In comparison to other studies in the CLC process, it was observed that the lifetime was greatly reduced as a consequence of the reducing conditions of the CLG process. Values of around 2000 h of the lifetime were found for iron ore after 50 h of operation in CLC conditions,^42^ much higher than the 300 h found in BCLG conditions. This is an important problem when the bed material is subjected to thermal stress and especially chemical stress by redox cycling under the highly reducing conditions of the CLG process. Nonetheless, no signs of agglomeration were observed in any of the ores.

**Figure 8:**
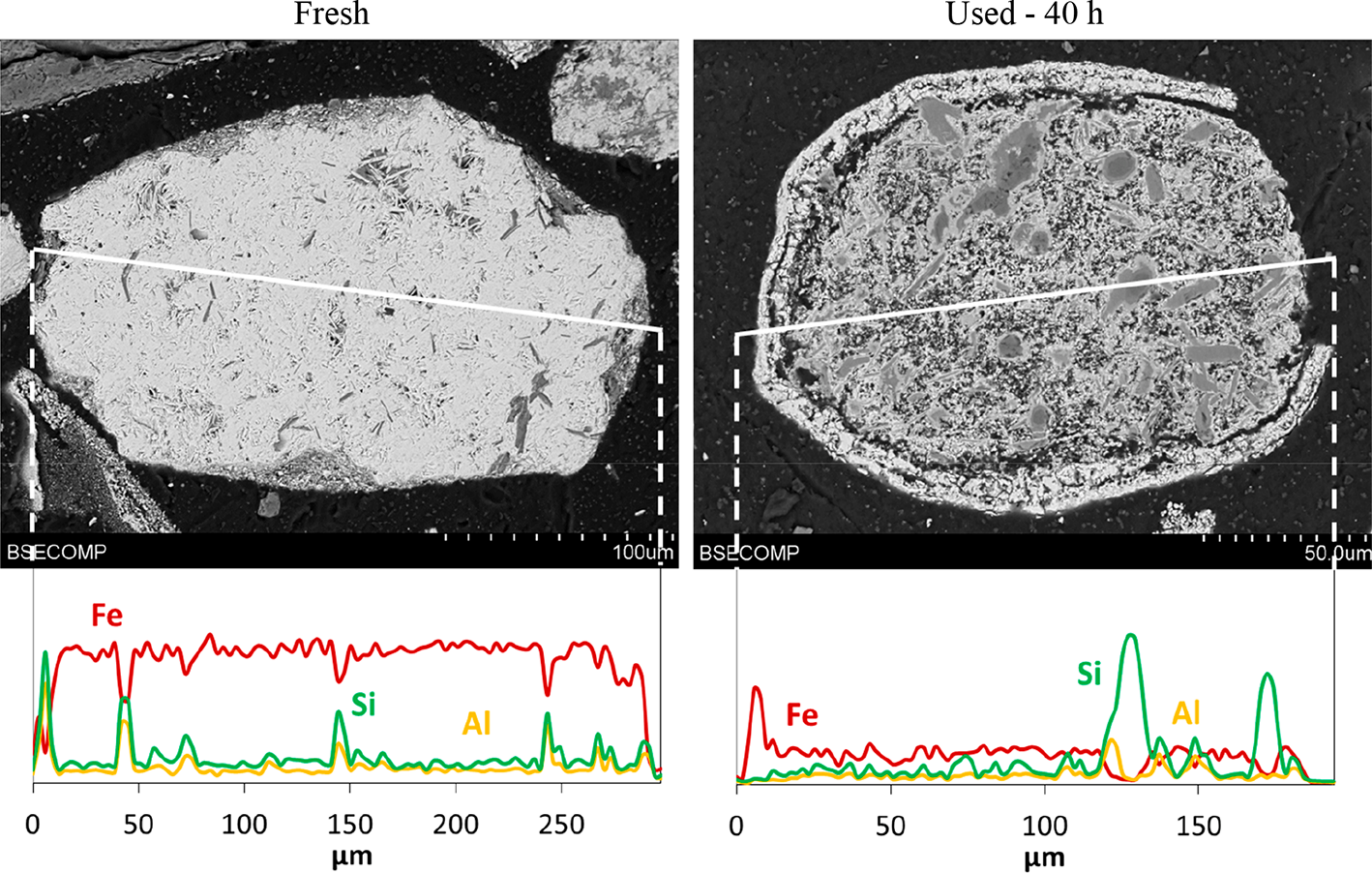
SEM–EDX images of iron ore particles before and after 40 h of continuous operation under CLG conditions.

**Figure 9:**
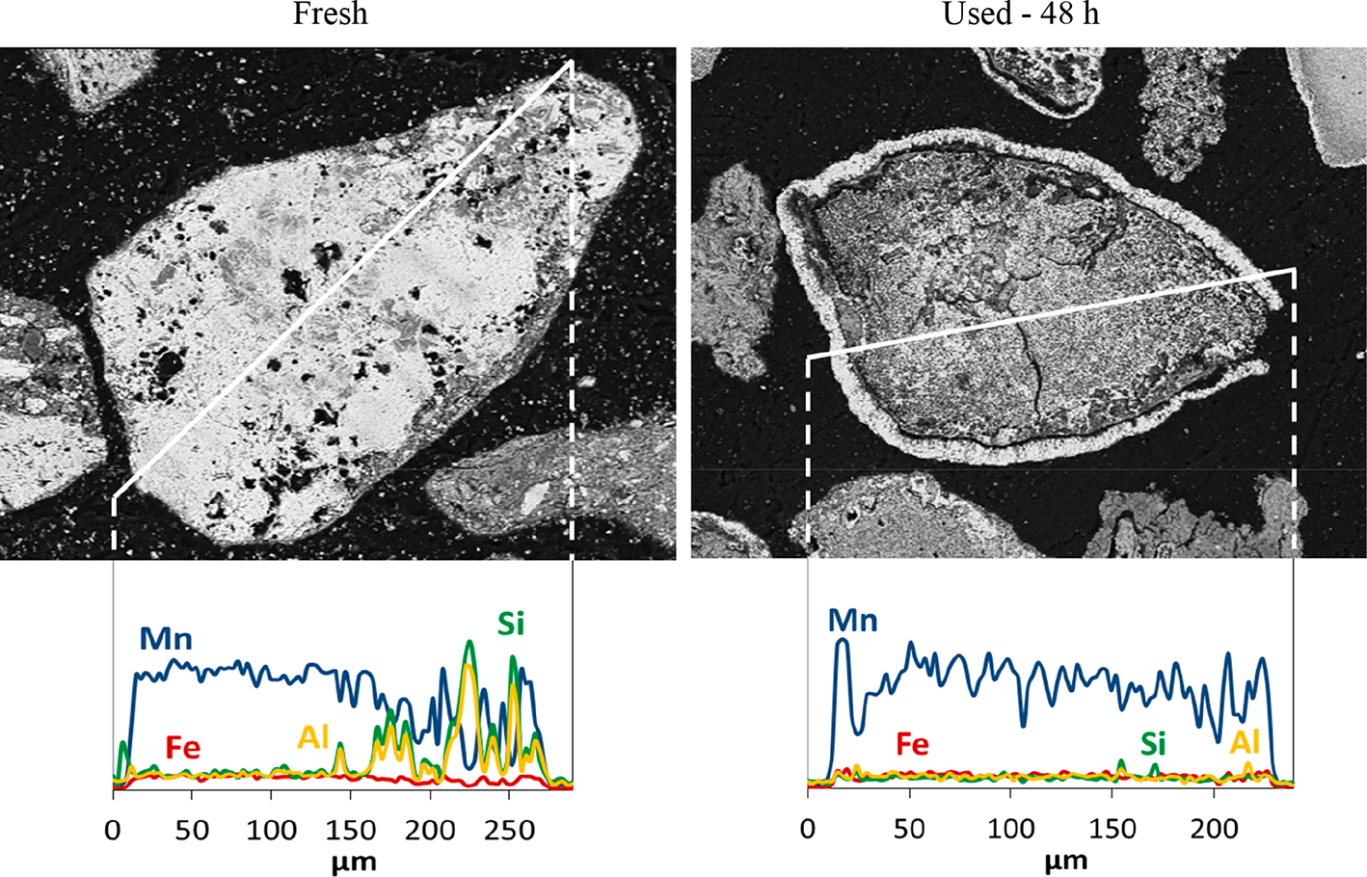
SEM–EDX images of Gabon manganese ore particles before and after 48 h of continuous operation under CLG conditions.

To perform the oxygen carrier characterization during the operating time under CLG conditions, particle samples were extracted from both reactors. The samples were analyzed with different techniques to observe the integrity and composition of the particles. Figures 8 and 9 show the pictures obtained of the internal structures of the iron ore and manganese ore samples by SEM–EDX analysis. The distribution of the main metals along the internal structure of the particles is also shown in the figures.

Note that the fresh particles of each ore, before being subjected to reducing conditions, had a compact structure with a good distribution of the major components, Fe in the case of the iron ore and Mn in the case of the manganese ore. According to other authors, it was also possible to observe the different phases present in the same particle.^43^

The samples obtained for each ore after completion of the CLG tests showed that, during the redox process, there was some migration of Fe and Mn toward the external surface of the particles for the iron and manganese ores, respectively. Thus, the formation of an external layer enriched in the mentioned metals is clearly visible, and in some cases, it was also observed that parts of the external shell broke away from the particle surface during the operation. This fact could affect the oxygen carrier transport capacity because part of the active phase is lost. In addition, the presence of cracks was observed. Besides that, the absence of agglomeration between particles was also confirmed.

**Figure 10:**
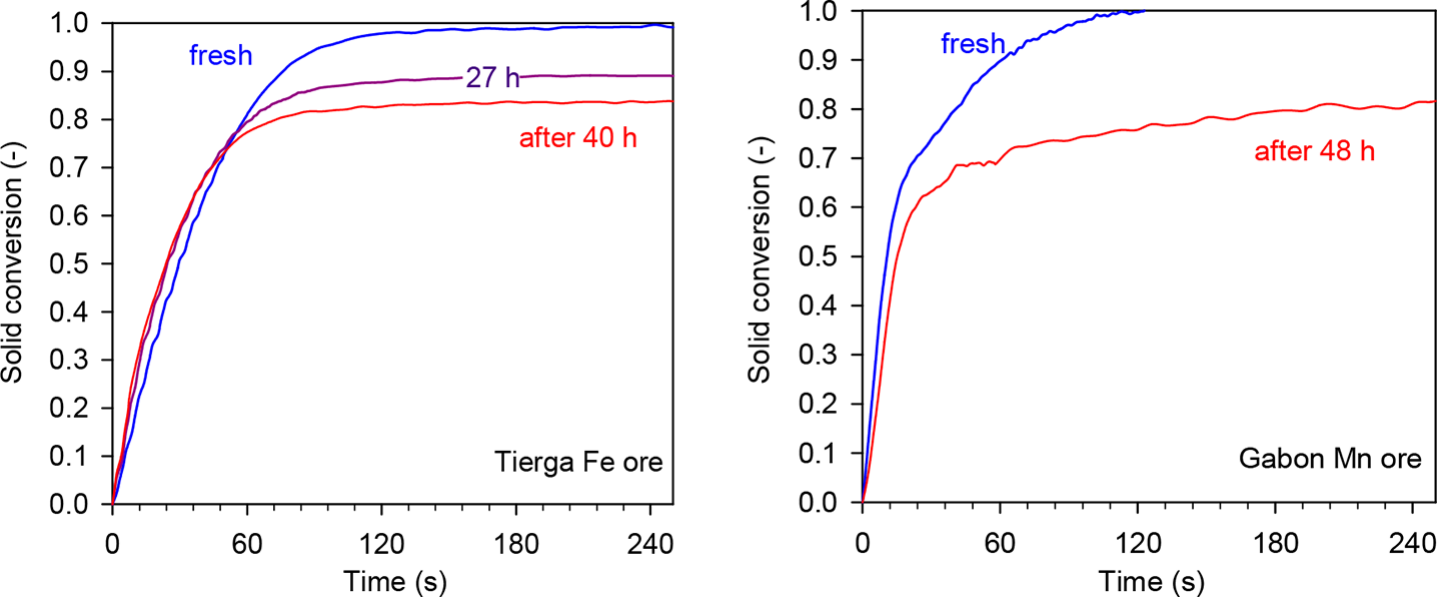
Conversion versus time curves for the reduction of iron and manganese ores as a function of the operation time. Reducing gas = 15 vol % H_2_ + 20 vol % H_2_O.

In comparison of both materials, it could be said that both suffer similar effects as a result of thermal, chemical, and physical stress. Even so, the difference in the lifetime observed above could be due to the greater occurrence of cracks in the Gabon ore particles or its lower initial crushing strength. Tests were performed with TGA using a mixture of 15 vol % H_2_ + 20 vol % H_2_O as the reducing agent and air for oxidation and to determine the evolution of reactivity and oxygen transport capacity of the materials. Figure 10 shows conversion versus time curves for the reduction of the iron and manganese ores. Fe_2_O_3_/FeO and Mn_3_O_4_/MnO were the pair redox considered for the calculation.

In good agreement with the SEM analysis, TGAs revealed that both materials suffered a gradual loss of the oxygen transport capacity, as a consequence of the migration of the active phases to the particle surface and the subsequent loss as a result of detachment and attrition. The iron ore showed a loss of the oxygen transport capacity from 7.7% of the fresh sample to 7.1% after 40 h of operation under CLG conditions. In the same way, the manganese ore showed a loss of the oxygen transport capacity from 5.6% of the fresh particles to 4.6% after 48 h of operation under CLG conditions.

The loss of the metals with redox activity was also demonstrated by ICP–OES analysis. The results showed that the iron ore lost about the 10 wt % of Fe present in the fresh material and the manganese ore lost about the 20 wt % of Mn present in the fresh material, while the amount of Fe remained constant. However, it has been observed that the loss of Fe and Mn in the different oxygen carriers did not affect the reactivity of the oxygen carriers in the majority of the conversion range, as shown in Figure 10.

### 3.6 Comparison of Minerals for BCLG

Most of the research on BCLG has been performed using iron-based oxygen carriers, such as iron oxides or ilmenite. Other investigations using hematite/magnetite as the redox pair were carried out using different experimental systems and ways of controlling the oxygen supplied to the fuel. In this section, a comparison among the performance of three oxygen carriers with data obtained in the same unit and using the same system of control of oxygen supplied to biomass in the fuel reactor was made. Ilmenite data were taken from Condori et al.^12^

**Figure 11:**
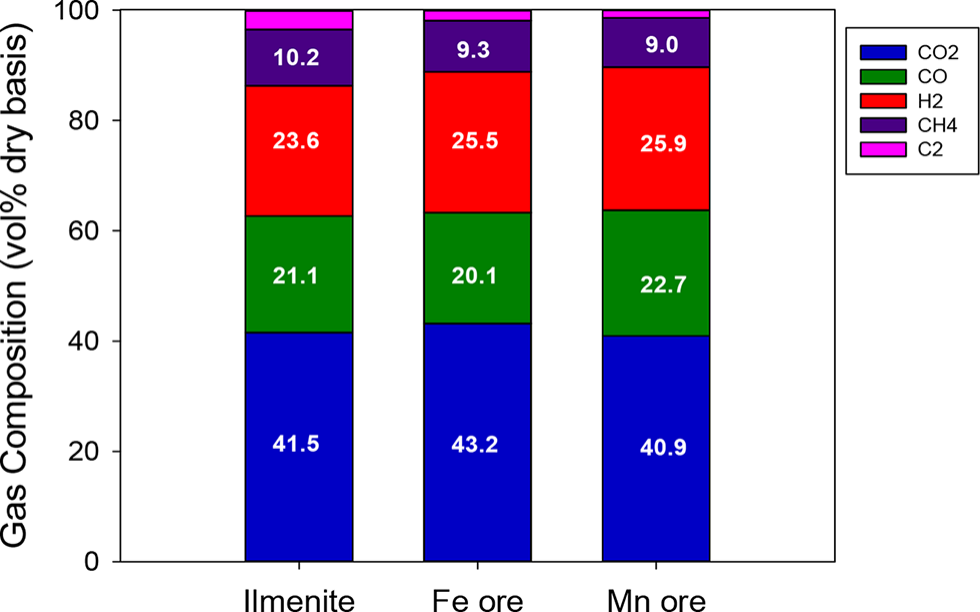
Comparison of the syngas composition obtained with the different oxygen carriers under autothermal conditions. λ = 0.35, S/B = 0.6, and *T*_FR_ = 940 °C.

**Figure 12:**
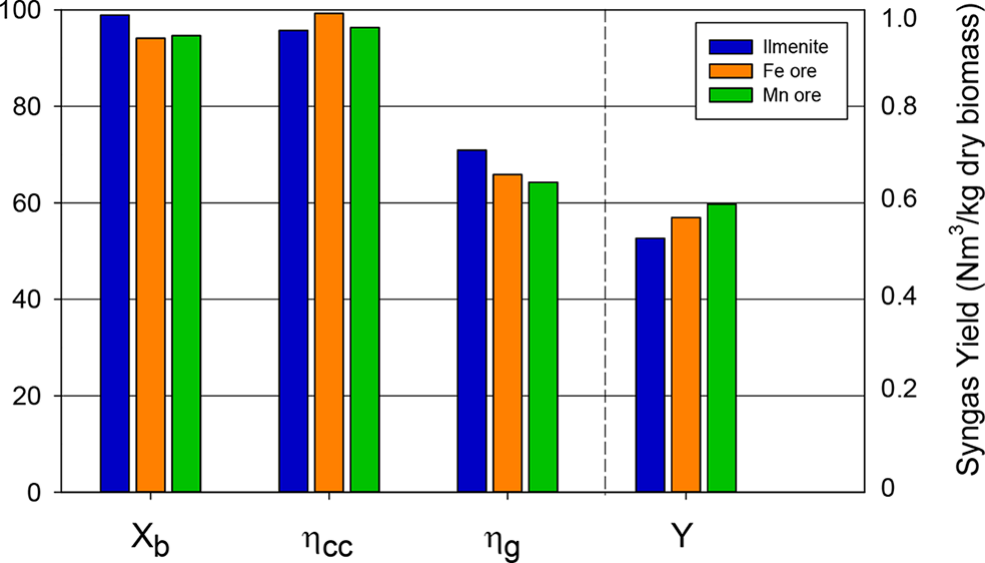
Comparison of the process efficiency parameters (%) obtained with the different oxygen carriers under autothermal conditions. λ = 0.35, S/B = 0.6, and *T*_FR_ = 940 °C.

Because experimental results were not obtained under identical operating conditions for all oxygen carriers, the experimental results were extrapolated to the same operating conditions of λ = 0.35, S/B = 0.6, and *T*_FR_ = 940 °C. These correspond approximately to autothermal conditions in a BCLG process.^41^Figures 11 and 12 show the results of syngas composition and process efficiencies that could be obtained under these conditions.

For the three oxygen carriers compared, biomass conversion (*X*_b_) was high, with values taken from 94.1% for iron ore to 98.9% for ilmenite. Moreover, carbon conversion efficiency (η_cc_) values were higher than 95% for all of the oxygen carriers, reaching a value of 99.3% for the iron ore. Cold gas efficiencies varied from ∼65% for iron and manganese ores to 70.9% for ilmenite, although the syngas yield was higher for iron and manganese ores than for ilmenite. As mentioned before, syngas yield differences can be attributed to the differences in the flow rates of CO and H_2_ generated by the reforming of CH_4_ and light hydrocarbons by the different ores. The main operating variable affecting the process and the syngas yield was the amount of oxygen supplied for biomass gasification. However, the most significant differences were found in the tar content in syngas and the lifetime of the oxygen carrier. Tar contents were 7 times higher for manganese ore than for ilmenite, indicating that the cleaning step for the manganese ore should be of greater intensity. The lifetime was very short for the manganese ore (160 h), while 600 h of the lifetime were estimated for ilmenite. It is necessary to mention that the lifetimes found in CLG were lower than those found in CLC and can be attributed to the deeper reduction of the oxygen carrier in the fuel reactor that was reduced to FeO, MnO, and FeTiO_3_ according with the determinations made on reduced samples taken in the fuel reactor. These lifetimes correspond to a higher cost for the makeup flow of the oxygen carrier lost by attrition in the process. The cost of minerals is approximately 200, 100, and 5.6 US$/tonne for ilmenite, iron ore, and manganese ore, respectively.^44^ Therefore, although the attrition for the Gabon Mn ore is very high, its low price could compensate for the need to replace the material more frequently.

For the selection of the most suitable oxygen carrier for the process, it would be necessary to consider the economy of the process taking into account the syngas composition, the syngas yield, the cost of tar cleaning and waste treatment, and the cost of the oxygen carrier makeup.

## 4 Conclusion

CLG of a wood residue was investigated in a continuous 1.5 kW_th_ unit, using as oxygen carriers an iron ore and a manganese ore during 40 and 48 h, respectively.

High biomass conversions (*X*_b_ > 94%) and carbon conversion efficiencies (η_cc_ > 95%) were found for both ores, showing the capability of the BCLG process to avoid CO_2_ emissions to the atmosphere.

The main variable affecting to the process performance parameters, such as the syngas yield and the cold gas efficiency, was the oxygen/biomass ratio, λ, which considers the amount of lattice oxygen supplied by the oxygen carrier for the biomass gasification. Small differences found in the performance of the different ores can be attributed to different degrees of CH_4_ and light hydrocarbons reforming in the process.

The fuel reactor temperature and the steam/biomass ratio (S/B) had a lower effect of the process performance and the syngas quality, although when the S/B ratio increased the ratio of H_2_/CO in the gas product also increased.

Syngas had a high CO_2_ content as a consequence of lattice oxygen supplied by the oxidized ore to generate by combustion the heat necessary for the gasification reactions to have an autothermal BCLG process. With the iron and manganese ores, CH_4_ contents of the order of 10% were found in syngas, coming from the unburnt or non-reformed volatiles.

Bed agglomeration was never found during the BCLG process with iron and manganese ores as oxygen carriers, although high attrition rates were obtained. Lifetimes of 160 and 300 h were inferred for the manganese and iron ores, respectively.

A migration of Fe or Mn to the external part of the particle was observed during operation in iron and manganese ores, which lead to an external shell highly concentrated on metal. Its detachment was responsible of the decrease in the oxygen transport capacity of the material with the operating time. The characterization of iron and manganese ores after operation allowed for the consideration of iron ore a suitable solid oxygen carrier for the BCLG process.

## Nomenclature

*F*_b_: flow of biomass fed into the system (kg/s)
*F*_b,dry_: flow of dry biomass fed into the system (kg/s)
*F*_C,AR,out_: molar flow of carbon in gases leaving the air reactor (kmol/s)
*F*_C,b_: molar flow of carbon in biomass fed into the fuel reactor (kmol/s)
*F*_C,FR,out_: molar flow of carbon in gases leaving the fuel reactor (kmol/s)
*F*_CO_2_,AR,out_: molar flow of carbon dioxide leaving the air reactor (kmol/s)
*F*_C,tar_: molar flow of carbon leaving the fuel reactor as tar (kmol/s)
*F*_g,FR,out_: molar flow of gas leaving the fuel reactor (kmol/s)
*F*_*i*_: molar gas flow of component *i* (kmol/s)
*F*_O_2_,AR,in_: molar flow of O_2_ fed into the air reactor (kmol/s)
*G*_H_2__: gas flow rate of H_2_ leaving the fuel reactor (Nm^3^/s)
*G*_CO_: gas flow rate of CO leaving the fuel reactor (Nm^3^/s)
LHV_b_: low heating value of the biomass (kJ/kg)
LHV_g_: low heating value of the produced gas (kJ/kmol)
*R*_oc_: oxygen transport capacity
*T*: temperature (°C)
*x*_*i*_: fraction of component *i* in the biomass
*X*_b_: biomass conversion
*Y*: syngas yield (Nm^3^/kg of dry biomass)
*Y*_CO_: CO yield (Nm^3^/kg of dry biomass)
*Y*_H_2__: H_2_ yield (Nm^3^/kg of dry biomass)
Ω_b_: oxygen demand for the complete combustion of the biomass (kmol of O/kg of biomass)
η_cc_: carbon conversion efficiency
η_g_: cold gas efficiency
λ: oxygen/biomass ratio
**Acronyms**
ASU: air separation unit
AR: air reactor
BCLG: biomass chemical looping gasification
BECCS: bioenergy with carbon capture storage
BET: Brunauer–Emmett–Teller
CLC: chemical looping combustion
CLG: chemical looping gasification
DFB: dual fluidized bed
DFBG: dual fluidized bed gasification
FR: fuel reactor
GC–MS: gas chromatography–mass spectrometry
GHG: greenhouse gas
ICP–OES: inductively coupled plasma optical emission spectroscopy
IWP: industrial wood pellet
LD: Linz–Donawitz
NDIR: non-dispersive infrared
OC: oxygen carrier
PSD: particle size distribution
SEM: scanning electron microscopy
TGA: thermogravimetric analysis
WGS: water–gas shift
XRD: X-ray diffraction
